# Turkish psychometric characteristics of the humanistic practice ability of nursing scale: differences by education, working year, and professional satisfaction

**DOI:** 10.1186/s12912-024-02083-9

**Published:** 2024-07-01

**Authors:** Ayse Sahin, Bedia Tarsuslu, Aslı Yilmaz, Filiz Kuni, Gulgun Durat

**Affiliations:** 1 Department of Child Care and Youth Services, Sabuncuoğlu Serefeddin Health Services Vocational School, Amasya University, Amasya, Türkiye Turkey; 2 Department of Psychiatric Nursing, Faculty of Health Sciences, Sakarya University, Sakarya, Türkiye Turkey; 3 Department of Child Health and Disease Nursing, Faculty of Health Science, Amasya University, Amasya, Türkiye Turkey; 4 Nurse, Sakarya Training and Research Hospital, Sakarya, Türkiye Turkey

**Keywords:** Humanism, Humanistic practice ability, Nursing, Validity-reliability

## Abstract

**Background:**

Humanistic nursing practices scientifically improve the knowledge structure of nursing, enrich its theoretical system and support its development. Therefore, it is crucial to evaluate the humanistic practice abilities of nurses.

**Objective:**

This study aimed to test the psycholinguistic features, language and construct validity of the Humanistic Practice Ability of Nursing Scale and to examine it according to nurses’ demographic characteristics.

**Design and methods:**

This study was a methodological type of analytical research conducted with 397 clinical nurses working in a hospital. A questionnaire including demographic information and evaluating empathy and compassion adequacy was used. Data were analyzed using explanatory and confirmatory factor analysis, Cronbach’s alpha, item-total score correlation, split-half analysis, t-test, analysis of variance and correlation analysis.

**Results:**

The scale consists of 29 items and four factors, explaining 61.15% of the total variance. Factor loads were > 0.30. confirmatory factor analysis results were χ2/df: 2.58, GFI: 0.86, TLI: 0.91, IFI: 0.92, CFI: 0.92, RMSEA: 0.06, and SRMR: 0.03. The Cronbach alpha value for the full scale is 0.95. A significant relationship was found between the scale and empathy and compassion proficiency. It was observed that the scale scores differed according to the nurses’ education level, working years and job satisfaction (*p* < 0.05).

**Conclusion:**

This study shows that the Turkish version of the HPAN scale is valid and reliable for 29 items and four factors. The humanistic practice ability of nurses differ according to postgraduate education, years of working in the profession and professional satisfaction.

**Supplementary Information:**

The online version contains supplementary material available at 10.1186/s12912-024-02083-9.

## Introduction

Nursing is a profession that is based on the concept of “valuing people” [1], emerged from the needs of society, protects and develops health and has a caring feature since its existence [2]. Nurses solve problems, think critically and determine care needs in line with the professional values of altruism, equality, aesthetics, freedom, human dignity, justice and truth [3]. While providing nursing care, it is necessary not to move away from humanism, which advocates respect for people’s beliefs, values, attitudes, individuality and rights [4]. Humanism in care; has been discussed by theorists such as Rogers, Paterson and Zderad, and is expressed as a nursing approach that provides common personality development in patients and nurses, improves the quality of care, improves the abilities and knowledge of caregivers, and supports their development [1]. In the Humanistic Nursing Theory explained by Paterson and Zderad, the way to respond to human needs has been tried to be explained, and it was emphasized that each nurse and patient is a unique human being, and their perspectives are equally important [5, 6]. It was reported that Rogers focuses on protecting health, preventing diseases, rehabilitating and providing humanistic care [7].

Humanism, a philosophical stance emphasizing humanity, has attracted the attention of researchers as well as theorists, and it has been the subject of many nursing studies [2, 6, 8–13]. França et al. [8] state that the humanistic nursing approach is accepted as a lively dialogue and consists of an intersubjective existential action. Humanistic Practice Ability of Nursing (HPAN) provides an objective reflection of the subjective attitudes of nurses as a part of the human spirit [12].

Wu and Volker [6] examined the relationship of Paterson and Zderad’s Humanistic Nursing Theory with hospice and palliative care nursing; they stated that this approach is a unifying language in planning care and defining interventions. In another study, it was reported that the humanistic nursing approach applied to individuals with coronary heart disease helps to alleviate clinical symptoms, eliminate negative emotions, improve body dysfunction and increase life quality [13]. In the result of Zamaniniya et al. [11] examined the results of humanistic nursing for intensive care nurses; they were stated that humanistic approaches in care play an essential role in meeting not only the needs of patients but also the personal and professional needs of nurses. In the findings of the same study, it was reported that humanistic nursing has outputs such as personal development, self-actualization, self-worth, and protection of personal dignity. Even humanistic nursing practices increase the popularity of nurses among their colleagues, patients, family members of patients and nursing managers [11]. In the case of ignoring humanistic nursing practices, conflicts are observed between nurses and patients [12]. Improving humanistic nursing practices contributes to more realistic and human-centred nursing and improves patient satisfaction and prognosis by improving health. It can also reduce medical expenses [14]. Moreover, HPAN is the primary indicator of quality in clinics and increases the quality-of-care nurses provide [15]. HPAN scientifically develops the knowledge structure of nursing, enriches its theoretical system and supports its development. Therefore, it is crucial to evaluate humanistic practice abilities in nurses.

Zhang et al. [12] developed the Humanistic Practice Ability of Nursing (HPAN) Scale is a mature and straightforward measurement tool that evaluates the complex structure of HPAN. This scale involves communication, psychological adaptation, ethical and legal practices, nursing aesthetics and practical care ability in nursing. The model on which they work contains the internalization process of humanistic nursing, which plays a bridge role in enabling the research of communication ability [8] and other sub-abilities in nursing. Each sub-ability is closely related to the quality of nursing. These five dimensions of the HPAN scale affect each other and act together. Maintaining coordination among these five sub-abilities is necessary to improve HPAN. In short, HPAN can develop nursing practices with a holistic and systematic perspective when these sub-abilities are taken into account. However, there needed to be a more valid and reliable measurement tool to evaluate nurses’ humanistic practice abilities in Turkey objectively. Yanmış et al. [16] tested the validity and reliability of the HPAN scale on Turkish nurses. However, when the results of this study are examined, it is seen that there are methodological problems in the process of evaluating the validity and reliability. The translation process from English to Turkish was carried out to ensure the language validity of the HPAN scale. However, Zhang et al. [12] developed a scale for Chinese-speaking nurses. In the studies of adapting the measurement tools from one language to another, translation and adaptation to the target language should be carried out through the form in the language in which the original data were collected [17, 18]. Ensuring language validity is the most crucial indicator of intelligibility and applicability in the target population in scale adaptation studies [17, 18]. Therefore, this study aimed to retest the psycholinguistic features, language, and construct validity of the HPAN scale and to examine it according to nurses’ demographic characteristics. In this study, in addition to the study of Yanmış et al. [16], the concordance validity of the scale was also tested. In addition, the average score differences between demographic characteristics in the test creation process were also examined.

*Main research questions*:

1. Is the Turkish version of the HPAN scale a valid and reliable measurement tool?
2. Do the HPAN scale scores of nurses differ according to their education, working year, and professional satisfaction?

## Materials and methods

The current research is analytical research of methodological type.

### Original HPAN scale

The Humanistic Practice Ability of Nursing Scale was developed to evaluate nursing communication, psychological adjustment, ethical and legal application, nursing aesthetics and caring practical abilities by Zhang et al. [12]. The scale is in the original development research, twenty-nine items, 5-point Likert type. Participants score each item as 1 = strongly disagree, 2 = disagree, 3 = undecided, 4 = agree, 5 = strongly agree. HPAN scale consists of five subscales representing humanistic nursing abilities. These are nursing communication ability, psychological adjustment ability, ethics and legal application ability, nursing aesthetic ability, and caring practical ability—scores from the scale range from 29 to 145. The development study observed that the Cronbach alpha values of the item ranged from α = 0.87 to 0.99 and had good to excellent internal reliability [12].

### Translation and cross-cultural adaptation

#### Translation procedure

Permission was obtained from the authors who developed the scale before starting the research. The translation-back translation method was used to ensure language validity [17]. First, the scale was translated into Turkish by two translators who are independent of each other, whose mother tongue is Turkish, who speak Chinese, and who have a good command of Chinese culture. Later, the researchers reviewed the translations and the Turkish form was created. For back translation, two translators fluent in Chinese and Turkish independently translated the Turkish version of the created scale back into Chinese. These two translators did not know the original Chinese version of the scale. The translations were reviewed, and their compatibility was evaluated by comparing them with the expressions in the original form.

#### Language validity

The Davis technique was used to test the language validity of the scale, which was translated into Turkish [17]. The finalized Turkish form was sent to 11 specialists in the field of nursing for their evaluation. Then, for each item in the scale, the Content Validity Average (CVA) and the Content Validity Index (CVI) for the total scale were calculated [17]. Revisions were made in the scale expressions in line with expert suggestions. In the revision, the expression “patient” in the items was changed to “individual I serve”. Then, to test the statements’ intelligibility, the scale was administered to 15 nurses independent of the research population and meeting the inclusion criteria. After each participant completed the scale form, a 5 to 10-minute interview was conducted to determine whether they understood the content of the scale items and to discuss whether there was any content that was not understood. The evaluations and feedback of the participants on the items were recorded, and the Turkish form was finalized (see Appendix. Turkish version of HPAN Scale).

### Samples and setting

The study population consisted of 1318 nurses working in inpatient and outpatient clinics in a university training and research hospital between January and May 2022. Convenience sampling was used for sample selection from the universe [19]. Inclusion criteria are to graduate from nursing school (high school, associate degree, university level), actively work in the inpatient and outpatient units of the hospital (annual leave, paid / not on paid leave), and answer all of the survey questions. Informed consent was obtained from all participants participating in the study. In scale adaptation studies, it is suggested that the number of participants in the sample should be between five and ten times the number of scale items [17]. In this respect, it was aimed to reach 145–290 nurses. Four hundred twenty-three nurses answered the data collection form, and 26 participants were excluded from the study because they did not answer all the questions. The research was completed with the participation of 397 nurses.

### Data collection tools

#### Demographic information form

The form consists of a total of 7 questions regarding the participants’ age, gender, marital status, educational status, position, year of research in total, and professional satisfaction.

#### Turkish version of the HPAN SCALE

After the language validity was ensured, the HPAN scale was administered to the participants as in the original structure, with 29 items and a 5-point Likert scale. High scores on the scale indicate that nurses have high humanistic practice ability [12]. In the current study, it was seen that the HPAN scale has a structure of 29 items and four factors: Ethics and legal application ability in care (F1), caring practical and nursing aesthetic ability (F2), psychological adjustment ability (F3) and nursing communication ability (F4).

#### Toronto empathy questionnaire

The Toronto Empathy Questionnaire (TEQ) is a 5-point Likert type consisting of 16 items. In the study conducted by Totan, Doğan, and Sapmaz [20] to test the Turkish validity and reliability of the TEQ, the number of items on the scale was reduced to 13 due to cultural differences. The internal consistency reliability coefficient of the scale was found to be 0.79. The total score on the scale ranges from 13 to 65. A high score indicates a high level of empathy [20]. In this study, the Cronbach alpha coefficient was found to be 0.82. The TEQ was used to evaluate the congruent validity of the HPAN scale.

#### The Compassion Competence Scale

The Compassion Competence Scale (CCS) was developed by Lee and Seomun [21]. The scale consists of three sub-dimensions: communication, sensitivity and insight. The highest score that can be obtained from the scale is 5, and the lowest score is 1. As the score increases, the level of compassion adequacy increases. The Turkish validity and reliability of the scale were performed by Çiftçi and Aras [22] Cronbach’s alpha coefficients for the sub-dimensions of the scale were between 0.64 and 0.76; for the whole scale, it was determined to be 0.80. In this study, Cronbach’s alpha coefficient was found to vary between 0.81 and 0.93. The CCS was used to evaluate the congruent validity of the HPAN scale.

### Data collection

Researchers invited the nurses and explained the research’s purpose. Volunteer nurses were asked to answer the questions in the questionnaire independently after obtaining their informed consent. The questionnaire was left to the participants to answer at a time convenient for them and to be retrieved later. Participants answered questions anonymously. The researchers checked the answered questionnaire to ensure reliability. Then, the forms’ data entry, all questions answered, was done in the computer environment.

### Data evaluation

Data were analyzed by transferring them to IBM SPSS Statistics 23 and IBM SPSS AMOS 23 programs. While evaluating the research data, frequency distribution for categorical variables and descriptive statistics (mean, standard deviation) for numerical variables were used. The Pearson correlation coefficient was used to examine the relationship between numerical variables. Explanatory and confirmatory factor analysis for scale construct validity, Cronbach’s alpha, item-total score correlation and split-half analysis were used for reliability. One-Way ANOVA test were used to examine the scale mean score differences according to demographic characteristics. Obtained results were tested at *p* < 0.05 significance level.

## Results

### Sample characteristics

The participants were 86.6% female, 13.4% male, 53.7% single, and 46.3% married, and the mean age was 30.12 ± 7.56 (min:21, max:62). 75.6% of the nurses have a bachelor’s degree, 23.4% are 0–1 years in the profession, 33.2% are 1.01-5 years, and 43.3% have worked for 5.01 years or more. 47.9% of the nurses stated that they were satisfied with nursing as professional satisfaction (*see* Table 1).

**Table 1:** Comparison of the total score of HPAN Scale by education, working year, and professional satisfaction

Variable		*n*	%	x̄ ± Sd	F	*p*	Differences
Education	High school^a^	30	7.6	134.10 ± 14.24	2.744	0.043*	d > c (*p* = 0.028)
	Associate’s degree^b^	40	10.1	129.70 ± 14.48			
	Bachelor’s degree^c^	300	75.6	128.58 ± 13.99			
	Master’s degree^d^	27	6.8	134.52 ± 9.35			
Working years	0–1 year^a^	93	23.4	131.99 ± 11.75	6.609	0.002*	a > b(*p* = 0.004). c > b (*p* = 0.007)
	1.01-5 years^b^	132	33.2	126.03 ± 15.66			
	5.01 yaers and more^c^	172	43.3	130.85 ± 13.05			
Professional satisfaction	Satisfied^a^	190	47.9	131.71 ± 12.87	4.661	0.010*	a > b (*p* = 0.019)
	Neither satisfied nor dissatisfied^b^	156	39.3	127.64 ± 14.12			
	Dissatisfied^c^	51	12.8	127.08 ± 15.8			

### Validity

#### Language and content validity

As a result of the opinion of 11 experts for the content validity of CVI, statistically, the CVA value of each item varied between 0.95 and 1.00. Since the CVA values were found to be greater than 0.80, item inference was not made [17]. The CVI value for the total of the scale was 0.99 ± 0.023. In line with these results, it was seen that the HPAN scale provided content validity.

#### Construct validity

Explanatory factor analysis (EFA, principal component analysis with maximum variance rotation) and confirmatory factor analysis (CFA) were applied to the data set to evaluate the scale’s construct validity. It has been reported that the application of CFA is sufficient in the studies of adapting measurement tools from one culture to another [23]. Therefore, in our study, CFA was first performed on the data. As the results were not acceptable (χ2/df: 3.19, GFI: 0.80, TLI: 0.87, IFI: 0.88, CFI: 0.88, RMSEA: 0.07, and SRMR: 0.03. First, EFA analysis was performed, and it was seen that some items were in different sub-dimensions from the original structure. The literature states that CFA analysis should also be applied in this case [17]. Therefore, firstly, Bartlett’s test of sphericity and Kaiser-Mayer-Olkin (KMO) index were used to evaluate the applicability of factor analysis to the data set. Factors with eigenvalues > 1 were examined. KMO index was 0.953, and Bartlett’s test was significant (x^2^ = 7046,366; *p* < 0.01). Accordingly, it was seen that the sample size in the data set was sufficient for factor analysis. However, it is reported that applying CFA to a different sample than EFA is appropriate. When the scale adaptation studies in the literature are examined, there are applications in which EFA and CFA were applied to the same data set [24, 25]. Accordingly, EFA and CFA were performed on the same data set.

As a result of the EFA, it was seen that the scale was collected in four sub-dimensions. Factor 1 (ethics and legal application ability in care) contained eleven items, Factor 2 (caring practical and nursing aesthetic ability) included seven items, Factor 3 (psychological adjustment ability) contained six items, and Factor 4 (nursing communication ability) contained five items (Table 2). It was determined that these four scale factors explained 61.15% of the total variance. Factor 1 explained 22.46% of the total variance, Factor 2 explained 14.62%, Factor 3 explained 13.37%, and Factor 4 explained 10.70%. Factor loads in these four sub-dimensions of the scale ranged from 0.34 to 0.83 (Table 2).

**Table 2:** Exploratory factor analysis and standard regression coefficients

Subscale	Items		EFA		CFA	Item-total correlation	Cronbach’s alpha if item deleted	Cronbach’s alpha
		Factor load	Eigenvalue	Explained variance	Standard regression coefficients			
F1	17	0.774	6.513	22.457	0.772	0.649	0.947	0.933
	15	0.772			0.796	0.718	0.947	
	14	0.771			0.733	0.640	0.948	
	23	0.711			0.838	0.742	0.947	
	21	0.709			0.774	0.669	0.947	
	27	0.704			0.779	0.687	0.947	
	19	0.697			0.824	0.732	0.947	
	13	0.661			0.727	0.717	0.947	
	12	0.636			0.700	0.700	0.947	
	18	0.546			0.703	0.666	0.947	
	24	0.463			0.598	0.577	0.948	
F2	28	0.721	4.241	14.624	0.677	0.562	0.948	0.849
	25	0.721			0.788	0.650	0.947	
	29	0.718			0.769	0.637	0.947	
	22	0.638			0.773	0.700	0.947	
	20	0.633			0.526	0.474	0.950	
	26	0.595			0.827	0.721	0.947	
	16	0.341			0.479	0.473	0.949	
F3	10	0.826	3.878	13.371	0.674	0.545	0.948	0.853
	11	0.754			0.629	0.540	0.948	
	9	0.678			0.749	0.617	0.948	
	7	0.588			0.689	0.588	0.948	
	8	0.577			0.681	0.609	0.948	
	6	0.421			0.716	0.612	0.948	
F4	2	0.793	3.102	10.695	0.724	0.524	0.949	0.805
	3	0.739			0.697	0.630	0.947	
	1	0.585			0.791	0.638	0.947	
	4	0.550			0.576	0.490	0.949	
	5	0.546			0.660	0.600	0.948	
Total Cronbach’s alpha								0.949

Total explained variance = 61.147

Kaiser-Meyer Olkin (KMO) = 0.953

Bartlett’s test x^2^ value 7046.366; *p* < 0.01

Note. EFA: exploratory factor analyses. CFA: confirmatory factor analyses

**Fig. 1:**
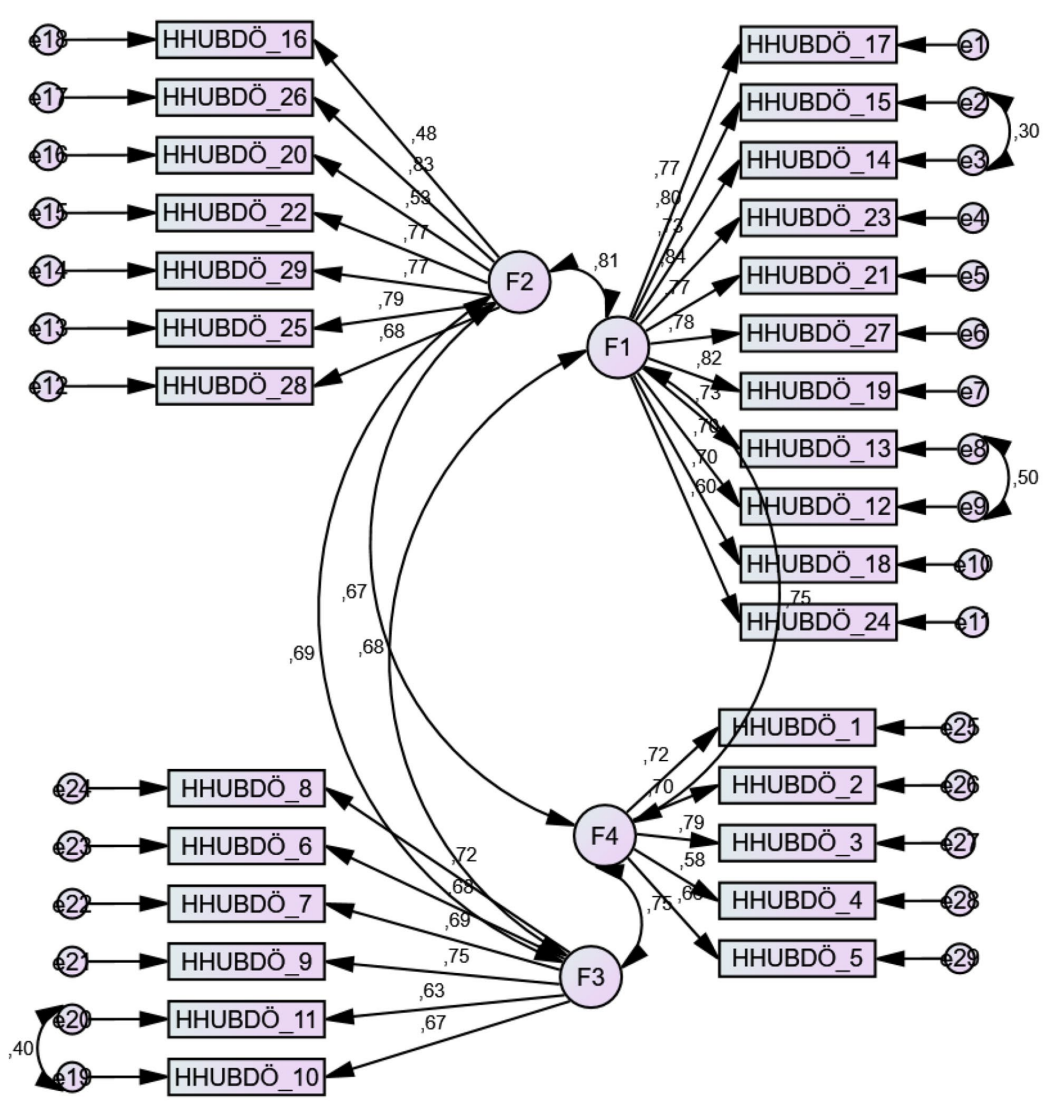
First-order CFA model of HPAN scale with four subscales. *Note.* HHUBDÖ: HPAN Scale

Then, CFA was performed to test the structure obtained from EFA. *The maximum likelihood method* was used for parameter estimation, and good fit index values were evaluated. CFA showed that the first model was poorly fit. In order to improve the compatibility indices, a two-way relationship was established between the error terms of the items with the highest modification indices value (10th-11th, 12th-13th and 14th-15th items). In addition, a relational setup between the factors was made to determine the expected covariance between the dimensions (Fig. 1). After three revisions, CFA results showed that χ2/df: 2.58, GFI: 0.86, TLI: 0.91, IFI: 0.92, CFI: 0.92, RMSEA: 0.06, and SRMR: 0.03 had an acceptable model fit. The standardized regression coefficients of the scale ranged from 0.48 to 0.84 (Table 2).

#### Convergent and discriminant validity

Another way to test validity is the convergent validity method, which is based on the assumption that the measurement tool is significantly related to a similar measurement tool that investigates the same concept or other parameters similar to itself and the original [23]. The convergent validity of the HPAN scale was tested using the TEQ and CCS. When the scale correlations were examined, a statistically significant positive correlation was found between the HPAN scale and TEQ (*r*=-0.480, *p* < 0.001) and CCS (*r*=-0.388, *p* < 0.001) (Table 3).

**Table 3:** Correlations between scales

		HPAN Scale					Compassion Competence Scale	Toronto Empathy Questionnaire
		F1	F2	F3	F4	Total		
HPAN scale	F1	1	0.70*	0.61*	0.65*	0.91*	0.31*	0.51*
	F2		1	0.62*	0.56*	0.85*	0.37*	0.36*
	F3			1	0.62*	0.82*	0.37*	0.32*
	F4				1	0.80*	0.27*	0.41*
	Total					1	0.39*	0.48*

Note. * *p* *< 0.05*

### Reliability

#### Item analysis

Item analysis was used to test the suitability of the scale items. It was observed that the correlation coefficients for the total score of the HPAN scale’s item were between 0.47 and 0.74 (> 0.30). The Cronbach’s alpha value of one item from the scale after deletion was lower than the Cronbach’s alpha value of the full scale before the deletion (Table 3). For this reason, item extraction was not carried out.

#### Internal coefficient

The Cronbach’s alpha coefficient for the whole HPAN scale was 0.95. The Cronbach’s alpha coefficients of the sub-dimensions for factors first, second, third and fourth, respectively, were found to be 0.93, 0.85, 0.85 and 0.81. When the item-total score correlation coefficients for reliability were examined, it was seen that they ranged from 0.47 to 0.74 (Table 3). In addition, split-half analysis was applied, with Cronbach alpha 0.91 for the first half; Cronbach alpha for the second half was calculated as 0.92. The correlation coefficient between the first and second half was 0.80 (*p* < 0.05), the Spearman-Brown coefficient was 0.87, and the Guttman split-half value was 0.87 (Table 4).

**Table 4:** Split-half analysis

Subscale	First-half cronbach’s α	Second-half cronbach’s α	Spearman-brown	Guttman split-half	Correlation between split halves	x̄ ± Sd (Min-Max)
	0.913	0.918	0.870	0.869	0.796	
F1						49.56 ± 5.50 (25–55)
F2						29.58 ± 3.81 (17–35)
F3						24.63 ± 3.41 (12–30)
F4						21.64 ± 2.90 (5–25)
Total						125.41 ± 13.38 (64–145)

### Differences by education, working year, and professional satisfaction

When the total score of the HPAN scale was compared according to demographic characteristics, the mean score of nurses with a Master’s degree/PhD degree is statistically significantly lower than that of nurses with a bachelor’s degree. The mean score of nurses who have worked for 1.01-5 years was statistically significantly lower than those who worked for 0–1 years or 5.01 years or more (*p* < 0.05). In addition, the total score of the HPAN scale was significantly higher for those who stated their job satisfaction level as “satisfied” compared to those who stated “neither satisfied nor dissatisfied” (F: 4.66; p: 0.010) (Table 1).

## Discussion

The humanistic approach is at the core of nursing practices and constitutes the essence of care. The results of this study showed that the Turkish version of the HPAN scale was a valid and reliable measurement tool with its 29-item, 4-factor structure to evaluate nurses’ humanistic ability in care practices.

In the Turkish version of the HPAN scale, the CVA scores of the items were > .80, and the scale CVI > .90, as a result of the evaluation of 11 experts. Therefore, the scale showed excellent content validity [17]. Later, when the EFA and CFA results were examined for construct validity, it was seen that the Turkish version of the HPAN scale had satisfactory validity. The EFA results of the scale included ‘ethics and legal application ability in care (Factor 1: items 12–15, 17–19, 21, 23, 24 and 27)’, ‘Nursing care practices and aesthetic ability (Factor 2: items 16, 20, 22, 25, 26, 28, 29)’, ‘psychological adjustment ability (Factor 3: items 6–11)’ and ‘nursing communication ability (Factor 4: items 1–5)’. This structure of the scale was different from the results of Zhang et al. [12] and Yanmış et al. [16] because they found the HPAN scale in five dimensions. This difference may be due to the cultural values of the sample in which the research was conducted. In the research of Zhang et al. [12], it is seen that the items belonging to the ‘caring practical ability’ sub-dimension are included in Factor 1 (items 23, 24 and 27) and Factor 2 (items 25, 26, 28, 29). For this research sample, it can be said that care practice abilities are whole with aesthetic, legal and ethical practice abilities and do not separate from each other.

The fact that the variance explained in the multidimensional scale is > 40% indicates that the scale’s construct validity is strong [17]. In this study, EFA results showed that the four-factor scale explained more than 61% of the total variance. In the research of Zhang et al. [12] and Yanmış et al. [16], it was observed that the number of items on the scale did not change in the current study, but it was gathered in four factors and had a different structure. Statisticians state that for an item to be included in a scale, the factor load must be at least 0.30 [17, 26]. The EFA results showed that the factor loadings of the HPAN scale were similar to the factor loadings in the original scale. However, the results prove this four-factor Turkish structure is valid in the present sample since factor loads are ≥ 30.

The results of the CFA analysis, applied to evaluate the fit of the structure obtained as a result of EFA, showed that the factor structure of the HPAN scale was appropriate. The first model did not show an acceptable fit in this research. After adding error terms for the three item pairs in the final CFA model, the model fit indices were improved, and there was a clear causal relationship between items 10 and 11, items 12 and 13, and items 14 and 15. Therefore, a two-way relationship was established between these error terms. CFA results showed that the chi-square value divided by degrees of freedom is less than five, RMSEA and SRMR were less than 0.08, TLI, IFI, and CFI fit indices were more remarkable than 0.90, and GFI was 0.86. Furthermore, the standard regression coefficients of all items were revealed that it is more significant than 0.30 [17]. Results showed that it is different in structure from the five-factor results of Zhang et al. [12] and Yanmış et al. [16]. However, according to the current research results, it has been proven that these four factors fit well as first-level indicators of the Turkish form of the scale and have a good factor structure for the current sample.

When convergent validity was examined, it was seen that there was a significant correlation between all score types of the HPAN scale. Furthermore, a significant positive correlation existed between the HPAN scale and TEQ and CCS scores. The results showed that the scale has convergent and discriminant [17]. Yanmış et al. [16] did not use these tests in their study, so the results could not be compared. However, Zhang et al. [12] evaluated the convergent validity, and the results are in a way that supports each other. For this reason, it can be said that while the HPAN scale factors show features that may be related to empathy and compassion proficiency in nurses’ practices, they also capture structures that evaluate humanistic practice abilities.

Item-total score analysis shows to what extent the items in a scale are related to the scale or subscale and among themselves and whether they measure the variable to be measured [17, 26]. The correlation in the item-total score analysis is expected to be positive and above 0.20 [26]. When the item analysis results were evaluated, item-total correlations were acceptable. In addition, according to the results, when the item was deleted, it was observed that there was no change in the total Cronbach’s alpha value of the scale compared to Cronbach’s alpha value before the deletion process, and item inference was not made. When the studies of Zhang et al. [12] and Yanmış et al. [16] were analyzed, it was seen that the results were similar. Therefore, all 29 items were included in the scale.

In the split-half method used in this study, Cronbach’s alpha values of both halves were found to be > 0.70 [17]. The two halves also had a strong and significant relationship; Spearman-Brown and Guttman Split-Half coefficients were > 0.70 [17]. These results show that the scale has a high level of reliability and provides internal validity. However, Zhang et al. [12] and Yanmış et al. [16] did not use these tests in their study, so the results could not be compared.

It has been reported that there was a positive relationship between nurses’ education level and work experience and their nursing practices and care behaviours [27]. In addition, when the relevant literature was examined, it was seen that the perception and implementation of care behaviours are affected by factors such as knowledge, education [28] and the duration of working directly with patients [29, 30]. In this study, nurses with a postgraduate education had higher HPAN abilities than those with a bachelor’s degree and those who have worked for 5.01 years or more than those who have been working for less support the literature. In addition, nurses’ professional satisfaction being ‘satisfied’ increases their HPAN ability compared to being ‘neither satisfied nor dissatisfied’.

### Limitations

The limitations of this research are:

- The HPAN scale was a subjective measurement tool.
- Due to regional constraints, participants’ cultural views and homogeneity are limited in this study.
- The analysis was made on the same data since the data set had to be divided into two to apply EFA and CFA, and the sample size had to be increased.
- Future studies on nurses from different regions are needed to re-evaluate the psychometric properties of the HPAN scale in different samples of various cultures.

## Conclusion

This study showed that the Turkish version of the HPAN scale was valid and reliable for 29 items and four factors. The presented findings provided psychometric evidence to assess the scale’s HPAN abilities in Turkish nurses. Larger samples are needed to test the reliability, validity and functionality of the HPAN scale in nurses working in various cultures and regions. It was observed that the nurses’ HPAN abilities differed in terms of their postgraduate education, their years of working in the profession and their professional satisfaction. This scale is expected to be widely used in research that evaluates and develops nursing practice abilities.

### Implications for nurses

The Turkish version of the HPAN scale can be used to identify factors and mechanisms that evaluate nurses’ humanistic practice abilities in the fields of ethics and legal application in care (F1), caring practical and nursing aesthetic (F2), psychological adjustment (F3) and nursing communication (F4). For this reason, the scale will be a guiding tool in evaluating and improving nursing practices by identifying the mechanisms that affect the quality of nursing service. In addition, comparing the scale item scores according to some characteristics (e.g., a clinic of work, year of research, etc.) can determine the aspects that negatively affect humanistic nursing practices. In this direction, it will guide the development of targeted training programs by nursing managers to improve the competencies of nurses in this field.

### Electronic supplementary material

Below is the link to the electronic supplementary material.

Supplementary Material 1

## Data Availability

The datasets used and/or analysed during the current study available from the corresponding author on reasonable request.

## References

[1] Taghinezhad F, Mohammadi E, Khademi M, Kazemnejad A (2022). Humanistic care in nursing: Concept analysis using rodgers’ evolutionary approach. Iran J Nurs Midwifery Res.

[2] Subaşi T, Özbek Güven G (2022). Professional values of nurses and affecting factors. USCEH Dergisi.

[3] Altun İ. Etik Ve Değerler. In: Aştı A, Karadağ A, editors. Hemşirelik Esasları Hemşirelik Bilimi ve Sanatı Cilt 1. Akademi Basın Yayıncılık; 2014. p. 119.

[4] Çoban N, Eryiğit T, Dülcek S, Derya Beydağ K, Ortabağ T (2022). The place of artificial intelligence and robot technologies in the nursing profession. Fenerbahce Univ J Heal Sci.

[5] Kleiman S. Josephine Paterson and Loeratt Zderad’s humanistic nursing theory. In: Parker ME, Smith MC, editors. Nursing theories nursing practice. 3rd ed. F.A. Davis Company; 2014. pp. 338–48.

[6] Wu HL, Volker DL (2012). Humanistic nursing theory: application to hospice and palliative care. J Adv Nurs.

[7] Kaya H, Yalçın Atar N, Eskimez Z. Hemşirelik Model ve Kuramları. In: Aştı TA, Karadağ A, editors. Hemşirelik Esasları Hemşirelik Bilimi ve Sanatı Cilt 1. Akademi Basım Yayıncılık; 2014. p. 89.

[8] Franca J, da Costa S, Lopes M, da Nobrega M, de Franca I (2013). The importance of communication in pediatric oncology palliative care: focus on humanistic nursing theory. Rev Lat Am Enfermagem.

[9] Lee H, Seo K (2021). Validity and reliability of the Korean version of the humanism scale short form: a cross-sectional study. Nurs Open.

[10] Létourneau D, Goudreau J, Cara C (2022). Nursing students and nurses’ recommendations aiming at improving the development of the humanistic caring competency. Can J Nurs Res.

[11] Zamaniniya Z, Khademi M, Toulabi T, Zarea K (2021). The outcomes of humanistic nursing for critical care nurses: a qualitative study. Nurs Midwifery Stud.

[12] Zhang J, Zhou X, Wang H, Luo Y, Li W (2021). Development and validation of the humanistic practice ability of nursing scale. Asian Nurs Res (Korean Soc Nurs Sci).

[13] Wang W, Jin L, Han W, Yi S, Hou X, Zhao X (2020). Application of humanistic nursing in patients with coronary heart disease and its influence on recovery. Int J Clin Exp Med.

[14] Antonini M, Bellier-Teichmann T, O’reilly L, Cara C, Brousseau S, Weidmann J (2021). Effects of an educational intervention to strengthen humanistic practice on haemodialysis nurses’ caring attitudes and behaviours and quality of working life: a cluster randomised controlled trial. BMC Nurs.

[15] Resnick B (2010). The difference nurses are making to improve quality of care to older adults through the interdisciplinary nursing quality research initiative. Geriatr Nurs (Minneap).

[16] Yanmış S, Bahçecioğlu Turan G, Özer Z (2022). Turkish validity and reliability study of humanistic practice ability of nursing scale. Int J Clin Pract.

[17] Alpar R. Spor Sağlık ve Eğitim Bilimlerinden Örneklerle Uygulamalı İstatistik ve Geçerlik Güvenirlik SPSS’de Çözümleme Adımları İle Birlikte. 7th ed. 2022.

[18] Çapık C, Gözüm S, Aksayan S (2018). Intercultural scale adaptation stages, language and culture adaptation: updated guideline. Florence Nightingale Hemşirelik Derg.

[19] Coşkun R, Yıldırım E, Altunışık R (2020). Sosyal Bilimlerde Araştırma Yöntemleri (SPSS Uygulamalı).

[20] Totan T, Dogan T, Sapmaz F (2012). The toronto empathy questionnaire: evaluation of psychometric properties among Turkish university students. Egit Arastirmalari-Eurasian J Educ Res.

[21] Lee Y, Seomun G (2016). Development and validation of an instrument to measure nurses’ compassion competence. Appl Nurs Res.

[22] Çiftçi B, Aras GN (2021). Adaptation of the compassion competence scale to Turkish. Perspect Psychiatr Care.

[23] Alpar R (2021). Uygulamalı Çok Değişkenli Istatistiksel Yöntemler.

[24] Altundağ Y, Ayas T (2018). Scale of coping strategies with cyberbullying for teachers: validity and reliability study. Anadolu Psikiyatri Derg.

[25] Kamış GZ, Erden Aki ŞÖ, Yıldız Mİ, Doğan Varan H, Dolgun AB (2019). The validity and the reliability of Turkish version of the self-stigma of depression scale. Türk Psikiyatr Derg.

[26] Karagöz Y (2021). SPSS AMOS META Uygulamalı Biyoistatistik.

[27] Compton EK, Gildemeyer K, Reich RR, Mason TM (2019). Perceptions of caring behaviours: a comparison of surgical oncology nurses and patients. J Clin Nurs.

[28] Xue M, Sun H, Xue J, Zhou J, Qu J, Ji S (2023). Narrative medicine as a teaching strategy for nursing students to developing professionalism, empathy and humanistic caring ability: a randomized controlled trial. BMC Med Educ.

[29] Haryani A, Lukmanulhakim. Predictors of nurse’s caring behavior towards patients with critical illness. KnE Life Sci. 2019;12–22.

[30] Lechleitner R (2019). A study to assess home health nurses from a carative perspective. Hosp Top.

