# Supplementary material for: Turkish psychometric characteristics of the humanistic practice ability of nursing scale: differences by education, working year, and professional satisfaction
Source: BMC Nurs. 2024 Jul 1;23:448. doi: 10.1186/s12912-024-02083-9 (PMC11218182; doi:10.1186/s12912-024-02083-9)
Supplement: Supplementary file 1 — Supplementary Material 1 [file 12912_2024_2083_MOESM1_ESM.docx]

**Appendix** Turkish version of HPAN Scale

**Hümanistik Hemşirelik Uygulama Becerilerini Değerlendirme Ölçeği**

Lütfen çalışma durumunuza en uygun olan seçeceği işaretleyiniz. Puanınız ne kadar yüksek olursa uygunluk dereceniz de o kadar yüksek olur.

|  | **İfadeler** | **Çok Uygun** | **Uygun** | **Kısmen** | **Uygun Değil** | **Hiç Uygun Değil** |
| --- | --- | --- | --- | --- | --- | --- |
| 1 | Hizmet sunduğum bireylerle iletişime geçerken anlaşılması kolay bir dil kullanırım. | 5 | 4 | 3 | 2 | 1 |
| 2 | Hizmet sunduğum bireylerle iletişime geçerken göz teması, jest ve mimikler gibi sözsüz iletişim yöntemlerini kullanırım. | 5 | 4 | 3 | 2 | 1 |
| 3 | Özel durumlarda, duruma uygun sözlü ve sözsüz iletişim yöntemlerini kullanmayı tercih ederim. | 5 | 4 | 3 | 2 | 1 |
| 4 | Uygun olmayan iletişim tekniklerinden kaçınırım (Aniden konu değiştirme, uygun olmayan güvence verme gibi). | 5 | 4 | 3 | 2 | 1 |
| 5 | Etkili iletişim kurabilmek için çeşitli iletişim yöntemlerini esnek biçimde kullanabilirim. | 5 | 4 | 3 | 2 | 1 |
| 6 | Hizmet sunduğum bireylerin memnuniyetsizliğini zamanla iletişim kurarak çözerim. | 5 | 4 | 3 | 2 | 1 |
| 7 | Başkalarıyla iyi kişilerarası ilişkiler kurarım. | 5 | 4 | 3 | 2 | 1 |
| 8 | Hemşirelik mesleğindeki zorluklara ve aksaklıklara doğru şekilde çözüm bulmaya çalışırım. | 5 | 4 | 3 | 2 | 1 |
| 9 | Çalışırken yaşadığım çatışmalarla doğru ve olumlu bir şekilde yüzleşirim. | 5 | 4 | 3 | 2 | 1 |
| 10 | Duygularımı uygun bir şekilde ifade ederim. | 5 | 4 | 3 | 2 | 1 |
| 11 | Duygularımı etkili bir şekilde kontrol ederim. | 5 | 4 | 3 | 2 | 1 |
| 12 | Hemşirelik yaparken mesleki etik ilkelere uyarım. | 5 | 4 | 3 | 2 | 1 |
| 13 | İşyerinde etik konularla ilgili makul kararlar veririm. | 5 | 4 | 3 | 2 | 1 |
| 14 | Hizmet sunduğum bireylerin mahremiyetini korumaya özen gösteririm. | 5 | 4 | 3 | 2 | 1 |
| 15 | Hemşirelik mesleğini icra ederken bilinçli ve ölçülü davranırım. | 5 | 4 | 3 | 2 | 1 |
| 16 | Hemşirelik bakımını planlarken bireyin menfaatini ön planda tutarım. | 5 | 4 | 3 | 2 | 1 |
| 17 | Hemşirelik yaparken yasalara ve kurallara uyarım. | 5 | 4 | 3 | 2 | 1 |
| 18 | Hizmet sunduğum bireylerin yasal hak ve çıkarlarının ihlalini önlemek için çabalarım. | 5 | 4 | 3 | 2 | 1 |
| 19 | Mesleğimi yaparken çizdiğim imaja özen gösteririm. | 5 | 4 | 3 | 2 | 1 |
| 20 | Hizmet sunduğum bireylerle iletişim kurarken sanatsal bir dil kullanmaya özen gösteririm. | 5 | 4 | 3 | 2 | 1 |
| 21 | Çalışırken sorumluluk duygusunu yoğun olarak hissederim. | 5 | 4 | 3 | 2 | 1 |
| 22 | Hizmet sunduğum bireyler için psikolojik, sosyal ve fizyolojik açıdan rahat ve güzel bir ortamı sağlarım. | 5 | 4 | 3 | 2 | 1 |
| 23 | Hemşirelik uygulamalarını yerine getirirken bireylere saygı gösteririm. | 5 | 4 | 3 | 2 | 1 |
| 24 | Çalışırken kendimi başkalarının yerine koyarım. | 5 | 4 | 3 | 2 | 1 |
| 25 | Hizmet sunduğum bireylerin sağlık gereksinimlerini (psikolojik, fizyolojik, sosyal) aktif bir şekilde karşılarım. | 5 | 4 | 3 | 2 | 1 |
| 26 | Hizmet sunduğum bireylere bütüncül bakım ve destek sağlarım. | 5 | 4 | 3 | 2 | 1 |
| 27 | Hizmet sunduğum bireylere eşit davranırım. | 5 | 4 | 3 | 2 | 1 |
| 28 | Hizmet sunduğum bireylerin durumuna ve geleneklerine göre çok kültürlü hemşirelik bakımı veririm. | 5 | 4 | 3 | 2 | 1 |
| 29 | Hizmet sunduğum bireylerin gereksinimlerini anlarım. | 5 | 4 | 3 | 2 | 1 |
